# Vitamin B_12_ and Semen Quality

**DOI:** 10.3390/biom7020042

**Published:** 2017-06-09

**Authors:** Saleem Ali Banihani

**Affiliations:** Department of Medical Laboratory Sciences, Jordan University of Science and Technology, Irbid 22110, Jordan; sabanihani@just.edu.jo; Tel.: +962-2720-1000

**Keywords:** vitamin B_12_, cobalamin, semen quality, sperm

## Abstract

Various studies have revealed the effects of vitamin B_12_, also named cobalamin, on semen quality and sperm physiology; however, these studies collectively are still unsummarized. Here, we systematically discuss and summarize the currently understood role of vitamin B_12_ on semen quality and sperm physiology. We searched the Web of Science, PubMed, and Scopus databases for only English language articles or abstracts from September 1961 to March 2017 (inclusive) using the key words “vitamin B_12_” and “cobalamin” versus “sperm”. Certain relevant references were included to support the empirical as well as the mechanistic discussions. In conclusion, the mainstream published work demonstrates the positive effects of vitamin B_12_ on semen quality: first, by increasing sperm count, and by enhancing sperm motility and reducing sperm DNA damage, though there are a few in vivo system studies that have deliberated some adverse effects. The beneficial effects of vitamin B_12_ on semen quality may be due to increased functionality of reproductive organs, decreased homocysteine toxicity, reduced amounts of generated nitric oxide, decreased levels of oxidative damage to sperm, reduced amount of energy produced by spermatozoa, decreased inflammation-induced semen impairment, and control of nuclear factor-κB activation. However, additional research, mainly clinical, is still needed to confirm these positive effects.

## 1. Introduction

Vitamin B_12_ (α-(5, 6-dimethylbenzimidazolyl) cobamidcyanide), also named cobalamin since it contains cobalt in the core of its molecular structure, is one of eight known B vitamins [1]. These vitamins are water-soluble and are essential for normal human growth, development, and metabolism [2]. In essence, vitamin B_12_ is synthesized by bacteria or archaea as they contain the required enzymes to assemble this molecular complexation [3]. Animal products such as meat, fish, and dairy products are proven food sources of vitamin B_12_ [4].

Vitamin B_12_ is involved in the metabolism of almost all cells in the human body as it is required for DNA synthesis, as well as amino acid and fatty acid metabolism [5]. Therefore, a wide symptomatic spectrum is related to vitamin B_12_ deficiency ranging from fatigue and depression to severe anemia and memory loss [6,7,8].

In many cases, vitamin B_12_ deficiency is associated with gastric achlorhydria (absence or reduced hydrochloric acid in the gastric secretions), resulting in decreased availability of intrinsic factor, a protein facilitates the absorption of vitamin B_12_ in the ileum [9]. Biochemically, vitamin B_12_ is considered as a coenzyme for methionine synthase enzyme [10,11]. This enzyme is required to synthesize methionine from homocysteine to complete the *S*-adenosylmethionine (SAM) cycle [12]. In this cycle, the critical step is the conversion of SAM to *S*-adenosylhomocysteine, which results in the methylation of the main functional macromolecules/molecules in the human body such as DNA, RNA, neurotransmitters, lipids, proteins, and amino acids [11,12].

In the last decade, various research studies worldwide have reported low levels or deficiency in vitamin B_12_ among the studied populations [13,14,15]. As a consequence, B_12_ supplementation has been recommended in many occasions as a key contribution to the maintenance and enhancement of population health [13,15,16]. Vitamin B_12_ deficiency was found to be multifactorial, and most of time it was related to malabsorption, malnutrition, or drug induced causes [17,18].

Since the beginning of the 60s, many studies (clinical and non-clinical) have investigated the effect of vitamin B_12_ on semen quality; this effect, however, has yet to be understood and summarized. This review systematically discusses and summarizes the up-to-date impact of vitamin B_12_ on semen quality and sperm physiology. To accomplish this, we searched the Web of Science, PubMed, and Scopus databases for only English language articles or abstracts from September 1961–March 2017 using the key words “vitamin B_12_” and “cobalamin” versus “sperm”. Furthermore, certain relevant references were included to support the empirical and mechanistic discussions.

## 2. Effect of Vitamin B_12_ on Sperm Parameters

### 2.1. Human Studies 

#### 2.1.1. Positive Effects

In humans, it has been found that vitamin B_12_ is transferred from the blood to the male reproductive organs, which emphasizes a substantial role of vitamin B_12_ in spermatogenesis, and hence in semen quality [19,20]. Supporting studies have shown that plasma vitamin B_12_ concentrations are lower in infertile men compared to fertile [21,22]. The positive effects of vitamin B_12_ on sperm parameters (e.g., count, motility, morphology, sperm DNA) have been investigated in various studies. Table 1 presents a summary of the human studies done on vitamin B_12_ and its derived compounds, and their reported positive effect on sperm count.

In 1984, Isoyama et al. [24] showed that methylcobalamin administered at 1500 µg/day to infertile, but not azoospermic, subjects, enhanced sperm motility by about 50% of cases after eight weeks of administration. Long-term treatment (>3 months) with methylcobalamin at 1500 µg/day increased sperm motility in patients with idiopathic oligozoospermia or normozoospermia [28]. In addition, the study by Boxmeer et al. [19] demonstrated a correlation between sperm count and vitamin B_12_ concentration in seminal plasma. A recent study by Gual-Frau et al. [27] showed that infertile men with varicocele administered multivitamin including vitamin B_12_ at 1 μg/day, for 3 months, had lower sperm DNA fragmentation by about 22.1%. 

Since 2000, a number of studies proposed vitamin B_12_ as a candidate therapy to recover or enhance semen quality. Sinclair [29] proposed vitamin B_12_ as a nutritional therapy that improved semen quality, mainly sperm count and motility. In 2006, vitamin B_12_ was suggested as one of the candidate drugs to manage male infertility due to its positive effects on sperm parameters, particularly sperm count [30]. In 2013, an oral antioxidant treatment including vitamin B_12_ was found to improve sperm vitality, motility, and DNA integrity [31]. Such evidence has allowed workers in the field to recommend the use of this antioxidants therapy prior to any assisted reproduction procedure (e.g., in vitro fertilization, intrauterine insemination), given that such intervention increases the success rate of fertilization. 

Despite the food sources of vitamin B_12_ being well-known (e.g., red meat, fish), only a few nutritional studies focusing on this area have been published. A study on an Indian population has shown that lactovegetarians from azoospermic, oligozoospermic, and normozoospermic subjects had mean values of seminal plasma vitamin B_12_ activity that were lower than the corresponding mean values in non-vegetarian subjects [32]. In the same study, azoospermic subjects were found to have lower levels of vitamin B_12_ when compared to oligozoospermic and normozoospermic [32]. However, vitamin B_12_ values in seminal plasma revealed no association with the sperm content of the corresponding semen in both oligozoospermic and normozoospermic subjects [32]. Another study on lactovegetarians supported the above findings by showing that these people had markedly lower levels of seminal vitamin B_12_ compared to non-vegetarians, while hydroxocobalamin treatment did not enhance the semen quality of oligozoospermic men with a low seminal content of vitamin B_12_ [33].

In general, amongst the infertile groups, infertile patients with varicocele have a higher proportion of sperm with damaged DNA [27,34]. A recent study by Gual-Frau et al. [27] showed that the integrity of sperm DNA in grade-I varicocele men can be improved by certain forms of oral multivitamin/antioxidant therapy, including vitamin B_12_.

#### 2.1.2. Negative Effects 

Only a few studies have presented the blunted effect of vitamin B_12_ on semen quality. A clinical trial in 1973 by Halim et al. [35] did not reveal a significant response of vitamin B_12_ therapy on semen quality. In this study, among 16 infertile patients injected weekly with cyanocobalamin for six weeks, only one patient had an improvement in sperm count, which was increased from 1 to 10 million mL^−1^. According to the authors, this patient may have suffered from vitamin B_12_ deficiency [35], while a more likely reason is that the result reflects the spontaneous variation in sperm concentration, which is sometimes observed in some individuals. Another study by Farthing et al. [36] did not find an obvious correlation between vitamin B_12_ levels and semen quality. Furthermore, a study by Chen et al. [37] demonstrated an insignificant difference in seminal vitamin B_12_ concentrations between fertile and infertile men (44 infertile vs. 176 fertile).

### 2.2. Rodent Studies

#### 2.2.1. Positive Effects 

Methylcobalamin at 1000 µg/kg (six times a week for 5–10 weeks) caused a marked increase in sperm count in oligospermically induced male rats [38]. In addition, oligozoospermic mice orally administered mecobalamin at 1.0 mg/kg/day for 10 weeks had a higher sperm count and more motile sperm, as well as lower sperm abnormalities compared with those of the control [39]. Moreover, methylcobalamin injected subcutaneously at 0.5 mg/kg (5 times per week) protected against ethylene oxide-induced testicular damage in male Wistar rates (higher sperm count, lower sperm abnormalities, and higher epididymis weight) [40]. Furthermore, methylcobalamin (1 mg/kg) protected against X-ray-induced testicular damage in male mice (higher sperm count motility and diameter of seminiferous tubules) [41].

Vitamin B_12_ deficiency significantly reduced the testes weight and induced clear morphological alterations in the testicular tissue of the B_12_-deficient mice [42]. Cimetidine, a member of the histamine-2 receptor (also called beta-blockers) antagonists family, was found to induce abnormal changes in the seminiferous tubules in the testis, which consequently negatively affects spermatogenesis, and hence semen quality [43]. B_12_ supplement was found to soften the detrimental effect of cimetidine on spermatogenesis and restore the number of Sertoli cells in adult male rates [44]. A recent study by Beltrame and Sasso-Cerri [45] revealed that vitamin B_12_ supplementation was able to recover cimetidine-induced sperm concentration. 

#### 2.2.2. Negative Effects 

Male rats fed a B_12_-deficient diet by pair-feeding for 100 days had atrophy in their seminiferous tubules and impaired spermatogenesis [46]. It has been suggested that dietary vitamin B_12_ deficiency affects both developing (damage to germ cells and sperm maturation) and growing (lower sperm count and morphology, but not motility) male rats [47]. In 2007, Watanabe et al. [47] showed that vitamin B_12_ deficiency during gestation and lactation phases affected the germ cells and led to spermatocyte destruction in the F1 male rats, which consequently reduced the quantity of the produced sperm. 

### 2.3. In Vitro Studies 

The addition of vitamin B_12_ at 2.50 mg/mL to bovine semen in vitro increased sperm motility, sperm velocity, and the number of plasma membrane-intact sperm compared to the control [48]. Bovine sperm cryoprotective medium supplemented with vitamin B_12_ at 2.50 mg/mL could reduce the freezing-thawing induced oxidative damage to sperm (e.g., higher catalase and glutathione reductase activities) [48]. Vitamin B_12_ at 2 mg/mL increased sperm parameters (viability, motility, progressive motility, and normality) of Dallagh rams in vitro in both pre- and post-freezing conditions [49]. 

## 3. Mechanistic Studies 

Vitamin B_12_ supplementation was found to be associated with marked histopathological improvements in the male reproductive system. For example, methylcobalamin at 1000 µg/kg (six times a week for 5–10 weeks) caused a marked increase in the diameter of the seminiferous tubules as well as sperm count in oligospermcally induced male rats [38]. In addition, oligozoospermic mice orally administered mecobalamin at 1.0 mg/kg/day for 10 weeks had a higher diameter of seminiferous tubules [39]. 

An in vivo system study conducted by Oh et al. [50] found that the transport of vitamin B_12_ in adult Leydig cells of the testes was mediated by the transmembrane protein amnionless, a protein that directs the endocytosis of cubilin, a receptor for the vitamin B_12_-intrinsic factor complex [50].

Only a few studies have presented the effects of vitamin B_12_ on gonadal function. Isoyama et al. [24] showed that infertile men administered methylcobalamin at 1.5 mg/day for 4–24 weeks had unchanged serum levels of testosterone, luteinizing hormone, or follicle-stimulating hormone [24].

Low levels of vitamin B_12_ in the body reduce the catalytic activity of methionine synthase to synthesize methionine from homoceysteine. This reduction leads to the accumulation of homocysteine in the plasma, also called hyperhomocysteinemia (~>15 µmol/L) [51]. Hyperhomocysteinemia has been found to be associated with various health problems, including reproductive disorders. For example, Ebisch et al. [52] demonstrated a significant inverse association between embryo quality following in vitro fertilization with intracytoplasmic sperm injection treatment and the total homocysteine concentration in seminal plasma. A recent in vitro study revealed a significant correlation between sperm parameters such as motility and count and thiol concentrations [53]. Such evidence suggests a possible homocysteine toxicity to sperm, which may negatively affect sperm parameters, as a result of reduced level of bodily vitamin B_12_. 

Homocysteine toxicity is mostly due to the reactive chemical structure of homocysteine, which contains a sulfhydryl group (thiol group) at one end and a carboxyl group at the other end. Chemically, the sulfur in the thiol group is very nucleophilic and can attack other molecular electrophiles [54].

One important set of homocysteine reactions in the body is the oxidation of thiol groups between homocysteine molecules and cysteine residues in other proteins to form disulfide bonds (also called disulfide bridges) [55,56]. Another set of bodily reactions of homocysteine is *N*-homocysteinylation. In this reaction, homocysteine thiolactone, an active cyclic thioester in which the carboxyl group is condensed with the sulfhydryl group, acylates the free amino groups of protein lysine residues [57]. The integration of homocysteine molecules with any given protein may significantly affect its functional domain, and therefore the entire protein function [58]. 

In the body, hyperhomocysteinemia was found to inhibit nitric oxide synthase pathways, which reduces the amount of nitric oxide produced [59]. Given that nitric oxide synthase is present in human spermatozoa [60], and that nitric oxide is crucial for adequate sperm motion [61], it is acceptable that vitamin B_12_ deficiency may reduce sperm function through hyperhomocysteinemia-induced nitric oxide depletion. 

Increased levels of reactive oxygen species in human semen has been found to increase oxidative injury to sperm, which negatively affects sperm quantity and quality [62]. Studies have revealed a negative correlation between vitamin B_12_ concentration and levels of reactive oxygen species in semen [37,63]. Accordingly, decreased levels of vitamin B_12_ may reduce semen quality as a result of increased accumulation of reactive oxygen species in semen and in reproductive organs.

The study by Hu et al. [48] indicated that the antioxidant activity of vitamin B_12_ prevents sperm membrane lipid-peroxidation during stress conditions such as freezing–thawing practices. Adding an appropriate amount of vitamin B_12_ into the freezing extender could prevent the generation of oxygen radicals, which decrease the damaging effect of lipid-peroxidation to sperm membranes, and ultimately improve sperm motility and viability [48].

In fact, a number of studies have revealed a powerful antioxidant activity for vitamin B_12_. Thiolatocobalamin was found to act as potent but benign antioxidant at pharmacological concentrations [64]. It was found that administration of vitamin B_12_ at 0.63 µg/kg/day for 30 days in combination with folic acid significantly reduced the arsenic-induced oxidative injury in rat pancreatic tissues [65]. In addition, cobalamin was found to protect against superoxide-induced cell injury in human aortic endothelial cells [66]. Moreover, in 2014, using the chemiluminescence method, Boyum et al. [67] showed that vitamin B_12_ had a significant reducing aptitude, which is the main chemical property of antioxidants. 

Creatine is naturally synthesized in the human body from the amino acids arginine and glycine [68]. In the first step of synthesis, these two amino acids are combined to produce guanidinoacetate [68]. Next, the latter is methylated, using SAM as the methyl donor, to produce creatine [68,69]. Given that vitamin B_12_ is crucial in synthesizing methionine [10], which is the precursor of SAM, the amount of SAM produced, and hence the amount of creatine, is affected by low levels of vitamin B_12_. In human spermatozoa, adenosine triphosphate (ATP) is generated from the chemical shuttle between creatine and creatine phosphate using creatine kinase [70]. Accordingly, it can be suggested that vitamin B_12_ deficiency alters sperm function by affecting the rapid buffering and regeneration of ATP. 

Systemic inflammation was found to be associated with low sperm count, abnormality of sperm morphology, and impaired sperm motility [71]. Evidence strongly suggests that vitamin B_12_ and transcobalamins supplementation may be beneficial in the management of systemic inflammatory response syndrome in some patients [72]. Such evidence suggests that vitamin B_12_ could be beneficial to decrease inflammation-induced semen impairment.

Studies have shown that, during testicular stress, Sertoli cell nuclear factor-κB proteins, transcription factors considered to be main regulators of the stress and immune responses, exert pro-apoptotic effects on germ cells, which consequently affect the number of sperm produced [73,74]. Vitamin B_12_ supplementation was found to be useful in controlling these transcription factors [72], thus avoiding the excessive germ cell death and hence sperm loss.

## 4. Conclusions and Future Perspectives

Thus far, the mainstream published studies (clinical, in vivo, or in vitro) present the positive effects (approximately 23 studies) of vitamin B_12_ on semen quality in primarily increasing sperm count and secondarily enhancing sperm motility and reducing sperm DNA damage, though there are still a few in vivo system studies (three studies) that have deliberated some adverse/blunted effects. As a result, vitamin B_12_, typically at the normal or therapeutic doses, is vital for adequate semen quality. 

The favorable effects of vitamin B_12_ on semen quality may be due to increased efficacy of male reproductive organs, decreased homocysteine toxicity, increased amount of nitric oxide produced, decreased accumulation of reactive oxygen species, reduced energy production by spermatozoa, decreased inflammation-induced semen impairment, and control of nuclear factor-κB activation.

However, further research—primarily clinical—is still necessary to confirm these favorable effects. At present, our laboratory is running a clinical study that uses different bioanalytical methods, such as flow cytometry, to standardize the favorable and unfavorable concentrations of vitamin B_12_ for human spermatozoa.

## Figures and Tables

**Table 1:** Effect of vitamin B_12_ or its derived compounds on human sperm count.

Affecter	Dose	Duration	Population	Effect on Sperm Parameters	Reference
**Vitamin B_12_ + Stilbestrol**	(Orally) (25 µg) B_12_ + (0.25 mg) stilbestrol	Daily, for 4 months.	Oligozoospermic patients; (*n* = 23)	(+) Sperm count	[23]
**Methylcobalamin**	1500 µg/day	4–24 weeks	Infertile men, excluding azoospermia	(+) Sperm count	[24]
**Methylcobalamin + Clomiphene Citrate (Clomid)**	(1500 µg/day) B_12_ + (25 mg/day) Clomid	12–24 weeks	Infertile men, excluding azoospermia	(+) Sperm count	[25]
**Methylcobalamin**	6000 µg/day	16 weeks	Oligozoospermic patients	(+) Sperm count	[22]
**Mecobalamin**	1500, 6000 µg/day	12 weeks	Oligozoospermic patients	(+) Sperm count	[26]
**Vitamin B_12_ + Other Antioxidants**	1 μg/day	3 months	Infertile men	(+) Sperm count	[27]
**Methylcobalamin**	1500 mg/day	>3 months	Patients with idiopathic oligozoospermia or normozoospermia	(+) Sperm count	[28]

(+): increase of parameter.

## References

[1] Adolfo F.R., do Nascimento P.C., Bohrer D., de Carvalho L.M., Viana C., Guarda A., Nunes Colim A., Mattiazzi P. (2016). Simultaneous determination of cobalt and nickel in vitamin B_12_ samples using high-resolution continuum source atomic absorption spectrometry. Talanta.

[2] Kennedy D.O. (2016). B vitamins and the brain: Mechanisms, dose and efficacy—A review. Nutrients.

[3] Fang H., Kang J., Zhang D. (2017). Microbial production of vitamin B_12_: A review and future perspectives. Microb. Cell Fact..

[4] Brouwer-Brolsma E.M., Dhonukshe-Rutten R.A., van Wijngaarden J.P., Zwaluw N.L., Velde N., de Groot L.C. (2015). Dietary sources of vitamin B-12 and their association with vitamin B-12 status markers in healthy older adults in the B-PROOF study. Nutrients.

[5] Lu H., Liu X., Deng Y., Qing H. (2013). DNA methylation, a hand behind neurodegenerative diseases. Front. Aging Neurosci..

[6] Dangour A.D., Allen E., Clarke R., Elbourne D., Fletcher A.E., Letley L., Richards M., Whyte K., Uauy R., Mills K. (2015). Effects of vitamin B-12 supplementation on neurologic and cognitive function in older people: A randomized controlled trial. Am. J. Clin. Nutr..

[7] Yadav M.K., Manoli N.M., Madhunapantula S.V. (2016). Comparative assessment of vitamin-B12, folic acid and homocysteine levels in relation to p53 expression in megaloblastic anemia. PLoS ONE.

[8] Tun A.M., Thein K.Z., Myint Z.W., Oo T.H. (2017). Pernicious anemia: Fundamental and practical aspects in diagnosis. Cardiovasc. Hematol. Agents Med. Chem..

[9] Oh R., Brown D.L. (2003). Vitamin B_12_ deficiency. Am. Fam. Phys..

[10] Liptak M.D., Datta S., Matthews R.G., Brunold T.C. (2008). Spectroscopic study of the cobalamin-dependent methionine synthase in the activation conformation: Effects of the y1139 residue and *S*-adenosylmethionine on the B_12_ cofactor. J. Am. Chem. Soc..

[11] Banerjee R.V., Matthews R.G. (1990). Cobalamin-dependent methionine synthase. FASEB J..

[12] Bottiglieri T., Laundy M., Crellin R., Toone B.K., Carney M.W., Reynolds E.H. (2000). Homocysteine, folate, methylation, and monoamine metabolism in depression. J. Neurol. Neurosurg. Psychiatry.

[13] Hinton C.F., Ojodu J.A., Fernhoff P.M., Rasmussen S.A., Scanlon K.S., Hannon W.H. (2010). Maternal and neonatal vitamin B_12_ deficiency detected through expanded newborn screening—United States, 2003–2007. J. Pediatr..

[14] Yaikhomba T., Poswal L., Goyal S. (2015). Assessment of iron, folate and vitamin B_12_ status in severe acute malnutrition. Indian J. Pediatr..

[15] Zhang D.M., Ye J.X., Mu J.S., Cui X.P. (2017). Efficacy of vitamin B supplementation on cognition in elderly patients with cognitive-related diseases. J. Geriatr. Psychiatry Neurol..

[16] Agnew-Blais J.C., Wassertheil-Smoller S., Kang J.H., Hogan P.E., Coker L.H., Snetselaar L.G., Smoller J.W. (2015). Folate, vitamin B-6, and vitamin B-12 intake and mild cognitive impairment and probable dementia in the women’s health initiative memory study. J. Acad. Nutr. Diet..

[17] Kancherla V., Elliott J.L., Patel B.B., Holland N.W., Johnson T.M., Khakharia A., Phillips L.S., Oakley G.P., Vaughan C.P. (2017). Long-term metformin therapy and monitoring for vitamin B_12_ deficiency among older veterans. J. Am. Geriatr. Soc..

[18] Hirschowitz B.I., Worthington J., Mohnen J. (2008). Vitamin B_12_ deficiency in hypersecretors during long-term acid suppression with proton pump inhibitors. Aliment. Pharmacol. Therapeut..

[19] Boxmeer J.C., Smit M., Weber R.F., Lindemans J., Romijn J.C., Eijkemans M.J., Macklon N.S., Steegers-Theunissen R.P. (2007). Seminal plasma cobalamin significantly correlates with sperm concentration in men undergoing IVF or ICSI procedures. J. Androl..

[20] Watson A.A. (1962). Seminal vitamin B_12_ and sterility. Lancet.

[21] Dhillon V.S., Shahid M., Husain S.A. (2007). Associations of MTHFR DNMT3b 4977 bp deletion in mtDNA and GSTM1 deletion, and aberrant CPG island hypermethylation of GSTM1 in non-obstructive infertility in indian men. Mol. Hum. Reprod..

[22] Moriyama H., Nakamura K., Sanda N., Fujiwara E., Seko S., Yamazaki A., Mizutani M., Sagami K., Kitano T. (1987). Studies on the usefulness of a long-term, high-dose treatment of methylcobalamin in patients with oligozoospermia. Hinyokika Kiyo.

[23] Goodhope C.D. (1961). The treatment of oligospermia with stilbestrol and vitamin B. Fertil. Steril..

[24] Isoyama R., Kawai S., Shimizu Y., Harada H., Takihara H., Baba Y., Sakatoku J. (1984). Clinical experience with methylcobalamin (CH3-B12) for male infertility. Hinyokika Kiyo.

[25] Isoyama R., Baba Y., Harada H., Kawai S., Shimizu Y., Fujii M., Fujisawa S., Takihara H., Koshido Y., Sakatoku J. (1986). Clinical experience of methylcobalamin (CH3-B12)/clomiphene citrate combined treatment in male infertility. Hinyokika Kiyo.

[26] Kumamoto Y., Maruta H., Ishigami J., Kamidono S., Orikasa S., Kimura M., Yamanaka H., Kurihara H., Koiso K., Okada K. (1988). Clinical efficacy of mecobalamin in the treatment of oligozoospermia—Results of double-blind comparative clinical study. Hinyokika Kiyo.

[27] Gual-Frau J., Abad C., Amengual M.J., Hannaoui N., Checa M.A., Ribas-Maynou J., Lozano I., Nikolaou A., Benet J., Garcia-Peiro A. (2015). Oral antioxidant treatment partly improves integrity of human sperm DNA in infertile grade Ivaricocele patients. Hum. Fertil..

[28] Iwasaki A., Hosaka M., Kinoshita Y., Saito K., Yumura Y., Ogawa T., Kan No H., Sato K. (2003). Result of long-term methylcobalamin treatment for male infertility. Jpn. J. Fertil. Steril..

[29] Sinclair S. (2000). Male infertility: Nutritional and environmental considerations. Altern. Med. Rev..

[30] Chatterjee S., Chowdhury R.G., Khan B. (2006). Medical management of male infertility. J. Indian Med. Assoc..

[31] Abad C., Amengual M.J., Gosalvez J., Coward K., Hannaoui N., Benet J., Garcia-Peiro A., Prats J. (2013). Effects of oral antioxidant treatment upon the dynamics of human sperm DNA fragmentation and subpopulations of sperm with highly degraded DNA. Andrologia.

[32] Jathar V.S., Hirwe R., Desai S., Satoskar R.S. (1976). Dietetic habits and quality of semen in indian subjects. Andrologia.

[33] Hirwe R., Jathar V.S., Desai S., Satoskar R.S. (1976). Vitamin B_12_ and potential fertility in male lactovegetarians. J. Biosoc. Sci..

[34] Ni K., Steger K., Yang H., Wang H., Hu K., Zhang T., Chen B. (2016). A comprehensive investigation of sperm DNA damage and oxidative stress injury in infertile patients with subclinical, normozoospermic, and astheno/oligozoospermic clinical varicocoele. Andrology.

[35] Halim A., Antoniou D., Leedham P.W., Blandy J.P., Tresidder G.C. (1973). Investigation and treatment of the infertile male. Proc. R. Soc. Med..

[36] Farthing M.J., Edwards C.R., Rees L.H., Dawson A.M. (1982). Male gonadal function in coeliac disease: 1. Sexual dysfunction, infertility, and semen quality. Gut.

[37] Chen Q., Ng V., Mei J., Chia S.E. (2001). Comparison of seminal vitamin B_12_, folate, reactive oxygen species and various sperm parameters between fertile and infertile males. Wei Sheng Yan Jiu.

[38] Ozaki S., Ohkawa I., Katoh Y., Tajima T., Kimura M., Orikasa S. (1988). Study on producing rats with experimental testicular dysfunction and effects of mecobalamin. Nihon Yakurigaku Zasshi.

[39] Oshio S., Ozaki S., Ohkawa I., Tajima T., Kaneko S., Mohri H. (1989). Mecobalamin promotes mouse sperm maturation. Andrologia.

[40] Mori K., Kaido M., Fujishiro K., Inoue N., Ide Y., Koide O. (1991). Preventive effects of methylcobalamin on the testicular damage induced by ethylene oxide. Arch. Toxicol..

[41] Oshio S., Yazaki T., Umeda T., Ozaki S., Ohkawa I., Tajima T., Yamada T., Mohri H. (1991). Effects of mecobalamin on testicular dysfunction induced by X-ray irradiation in mice. Nihon Yakurigaku Zasshi.

[42] Kawata T., Funada U., Wada M., Matsushita M., Sanai T., Yamada H., Kuwamori M., Arai K., Yamamoto Y., Tanaka N. (2004). Breeding severely vitamin B_12_-deficient mice as model animals. Int. J. Vitam. Nutr. Res..

[43] Banihani S.A. (2016). Histamine-2 receptor antagonists and semen quality. Basic Clin. Pharm. Toxicol..

[44] Beltrame F.L., Caneguim B.H., Miraglia S.M., Cerri P.S., Sasso-Cerri E. (2011). Vitamin B_12_ supplement exerts a beneficial effect on the seminiferous epithelium of cimetidine-treated rats. Cells Tissues Organs.

[45] Beltrame F.L., Sasso-Cerri E. (2016). Vitamin B_12_-induced spermatogenesis recovery in cimetidine-treated rats: Effect on the spermatogonia number and sperm concentration. Asian J. Androl..

[46] Kawata T., Tamiki A., Tashiro A., Suga K., Kamioka S., Yamada K., Wada M., Tanaka N., Tadokoro T., Maekawa A. (1997). Effect of vitamin B_12_-deficiency on testicular tissue in rats fed by pair-feeding. Int. J. Vitam. Nutr. Res..

[47] Watanabe T., Ebara S., Kimura S., Maeda K., Watanabe Y., Watanabe H., Kasai S., Nakano Y. (2007). Maternal vitamin B_12_ deficiency affects spermatogenesis at the embryonic and immature stages in rats. Congenit. Anom. (Kyoto).

[48] Hu J.H., Tian W.Q., Zhao X.L., Zan L.S., Xin Y.P., Li Q.W. (2011). The cryoprotective effects of vitamin B_12_ supplementation on bovine semen quality. Reprod. Domest. Anim..

[49] Hamedani M.A., Tahmasbi A.M., Ahangari Y.J. (2013). Effects of vitamin B_12_ supplementation on the quality of ovine spermatozoa. Open Vet. J..

[50] Oh Y.S., Park H.Y., Gye M.C. (2012). Expression of amnionless in mouse testes and leydig cells. Andrologia.

[51] Guo H., Chi J., Xing Y., Wang P. (2009). Influence of folic acid on plasma homocysteine levels and arterial endothelial function in patients with unstable angina. Indian J. Med. Res..

[52] Ebisch I.M., Peters W.H., Thomas C.M., Wetzels A.M., Peer P.G., Steegers-Theunissen R.P. (2006). Homocysteine, glutathione and related thiols affect fertility parameters in the (sub)fertile couple. Hum. Reprod..

[53] Kralikova M., Crha I., Huser M., Melounova J., Zakova J., Matejovicova M., Ventruba P. (2016). The intracellular concentration of homocysteine and related thiols is negatively correlated to sperm quality after highly effective method of sperm lysis. Andrologia.

[54] Schoneich C. (2017). Sulfur radical-induced redox modifications in proteins: Analysis and mechanistic aspects. Antioxid. Redox Signal..

[55] Malinowska J., Nowak P., Olas B. (2011). Comparison of the effect of homocysteine in the reduced form, its thiolactone and protein homocysteinylation on hemostatic properties of plasma. Thromb. Res..

[56] Zinellu A., Sotgia S., Scanu B., Pisanu E., Sanna M., Sati S., Deiana L., Sengupta S., Carru C. (2010). Determination of homocysteine thiolactone, reduced homocysteine, homocystine, homocysteine-cysteine mixed disulfide, cysteine and cystine in a reaction mixture by overimposed pressure/voltage capillary electrophoresis. Talanta.

[57] Jakubowski H. (2000). Calcium-dependent human serum homocysteine thiolactone hydrolase. A protective mechanism against protein n-homocysteinylation. J. Biol. Chem..

[58] Perla-Kajan J., Utyro O., Rusek M., Malinowska A., Sitkiewicz E., Jakubowski H. (2016). *N*-homocysteinylation impairs collagen cross-linking in cystathionine β-synthase-deficient mice: A novel mechanism of connective tissue abnormalities. FASEB J..

[59] Stuhlinger M.C., Tsao P.S., Her J.H., Kimoto M., Balint R.F., Cooke J.P. (2001). Homocysteine impairs the nitric oxide synthase pathway: Role of asymmetric dimethylarginine. Circulation.

[60] Lewis S.E., Donnelly E.T., Sterling E.S., Kennedy M.S., Thompson W., Chakravarthy U. (1996). Nitric oxide synthase and nitrite production in human spermatozoa: Evidence that endogenous nitric oxide is beneficial to sperm motility. Mol. Hum. Reprod..

[61] Miraglia E., De Angelis F., Gazzano E., Hassanpour H., Bertagna A., Aldieri E., Revelli A., Ghigo D. (2011). Nitric oxide stimulates human sperm motility via activation of the cyclic GMP/protein kinase G signaling pathway. Reproduction.

[62] Banihani S., Sharma R., Bayachou M., Sabanegh E., Agarwal A. (2012). Human sperm DNA oxidation, motility and viability in the presence of l-carnitine during in vitro incubation and centrifugation. Andrologia.

[63] Kifle L., Ortiz D., Shea T.B. (2009). Deprivation of folate and B_12_ increases neurodegeneration beyond that accompanying deprivation of either vitamin alone. J. Alzheimer’s Dis..

[64] Birch C.S., Brasch N.E., McCaddon A., Williams J.H. (2009). A novel role for vitamin B_12_: Cobalamins are intracellular antioxidants in vitro. Free Radical Biol. Med..

[65] Mukherjee S., Das D., Mukherjee M., Das A.S., Mitra C. (2006). Synergistic effect of folic acid and vitamin B_12_ in ameliorating arsenic-induced oxidative damage in pancreatic tissue of rat. J. Nutr. Biochem..

[66] Moreira E.S., Brasch N.E., Yun J. (2011). Vitamin B_12_ protects against superoxide-induced cell injury in human aortic endothelial cells. Free Radic. Biol. Med..

[67] Boyum A., Forstrom R.J., Sefland I., Sand K.L., Benestad H.B. (2014). Intricacies of redoxome function demonstrated with a simple in vitro chemiluminescence method, with special reference to vitamin B_12_ as antioxidant. Scand. J. Immunol..

[68] Brosnan M.E., Brosnan J.T. (2016). The role of dietary creatine. Amino Acids.

[69] Osna N.A., Feng D., Ganesan M., Maillacheruvu P.F., Orlicky D.J., French S.W., Tuma D.J., Kharbanda K.K. (2016). Prolonged feeding with guanidinoacetate, a methyl group consumer, exacerbates ethanol-induced liver injury. World J. Gastroenterol..

[70] Banihani S.A., Abu-Alhayjaa R.F. (2016). The activity of seminal creatine kinase is increased in the presence of pentoxifylline. Andrologia.

[71] Omu A.E. (2013). Sperm parameters: Paradigmatic index of good health and longevity. Med. Princ. Pract..

[72] Manzanares W., Hardy G. (2010). Vitamin B_12_: The forgotten micronutrient for critical care. Curr. Opin. Clin. Nutr. Metab. Care.

[73] Pentikainen V., Suomalainen L., Erkkila K., Martelin E., Parvinen M., Pentikainen M.O., Dunkel L. (2002). Nuclear factor-κB activation in human testicular apoptosis. Am. J. Pathol..

[74] Zhao Y., Kong C., Chen X., Wang Z., Wan Z., Jia L., Liu Q., Wang Y., Li W., Cui J. (2016). Repetitive exposure to low-dose X-irradiation attenuates testicular apoptosis in type 2 diabetic rats, likely via Akt-mediated Nrf2 activation. Mol. Cell. Endocrinol..

